# Allelopathy: Mechanisms and Applications in Regenerative Agriculture (2nd Edition)

**DOI:** 10.3390/plants14111565

**Published:** 2025-05-22

**Authors:** Margot Schulz, Vincenzo Tabaglio

**Affiliations:** 1 IMBIO Institute of Molecular Physiology and Biotechnology of Plants, University of Bonn, Karlrobert-Kreiten Str. 13, 53115 Bonn, Germany; 2 Department of Sustainable Crop Production DI.PRO.VE.S., Section Agronomy and Plant Biotechnologies, Università Cattolica del Sacro Cuore, Via Emilia Parmense 84, 29122 Piacenza, Italy; vincenzo.tabaglio@unicatt.it

Allelopathy is increasingly seen as a tool that can be used to reduce the overuse of synthetic herbicides and thus usher in an age of sustainable agriculture. The first edition of the Special Issue “Allelopathy: Mechanisms and Applications in Regenerative Agriculture” presented a collection of studies and reviews that mainly focused on identifying allelochemicals occurring in infrequently studied donor plants and the effects of these compounds on weeds and crops [1]. In response to the increasing interest in this topic, we have launched a second edition of this Special Issue. Once again, identifying bioactive and phytotoxic molecules remains an important issue. As stated before, microorganisms are critical in allelopathic interactions—a notion that was formerly neglected, aside from the roles of microorganisms in generating phytotoxic molecules from less bioactive precursors, and vice versa. However, studying the molecular background of detoxification processes and the microbial degradation of allelochemicals as a component of sustainability has become an important part of recent allelopathic research. With some exceptions, field studies are still in the minority, and the evaluation of shaping soil/plant microbiomes triggered by allelochemicals, as well as cover crops and mulches, has just begun. The rapidly growing interest in the role of secondary metabolites in modulating plant–microbe interactions is mirrored by recent studies, such as the multi-omics approach employed by Gao et al. [2], who elucidate the complex interactions between secondary metabolites and members of root-associated microbiome, or the work of Thoenen et al., who studied the cooperation of maize root bacteria in MBOA degradation [3].

The second edition of this Special Issue on allelopathy presents basic and applied research on allelochemicals, their effects on weeds and crops, and the contribution of microorganisms to allelopathic interactions. The seven research articles addressing different classes of allelochemicals (benzoxazinoids, phenoxazinones, phenolic acids, coumarins, cyclic isothiocyanate goitrin, glucosinolates, diketopiperazine, etc.) complement the first edition.

Some key points of the research articles are listed below. Weston et al. [4] evaluated the effects of several early-vigor genotypes on the production and release of targeted benzoxazinoids by field-grown wheat roots over a two-year period. The soil concentrations of the benzoxazolinone MBOA and several aminophenoxazinones differed greatly between years and among genotypes. Several microbially transformed aminophenoxazinones in the rhizosphere of many of the genotypes were observed.

Magedans et al. [5] studied the chemical composition and phytotoxic activity of an aqueous extract (AE) from *Myrciaria cuspidata* leaves. The extract, containing phenolic compounds, inhibited the activity of *Lactuca sativa* L. and the weed *Bidens pilosa* L., and it is suggested to be an environmentally friendly pre-emergence bioherbicide. Jeong et al. [6] dealt with the deleterious effects of the cyclic isothiocyanate goitrin on the nodule proteomes of the host *Lotus japonicus* and symbiotic *Mesorhizobium loti* and of free-living bacteria. The proteomes indicate a loss of immunity suppression resulting in the termination of symbiosis. Goitrin causes nodule dysfunction, failed nodule development, and N deficiency in the leaves. Krumsri et al. [7] investigated the allelopathic potential of sugarcane. Using an optimized organic-solvent extraction and fractionation protocol, they obtained fractions that selectively inhibited *Amaranthus viridis* and *Echinochloa crus-galli* in laboratory tests. The AE fraction exhibited early post-emergence activities. Moh et al. [8] explored allelopathy potential and allelochemicals in the leaves of the *Aegle marmelos* L. tree. Aqueous methanol extracts significantly inhibited the germination and growth of *Lepidium sativum*, *Lactuca sativa*, *Medicago sativa*, *Echinochloa crus-galli*, *Lolium multiflorum*, and *Phleum pratense*. Five active compounds were identified: umbelliferone, trans-ferulic acid, (E)-4-hydroxycinnamic acid methyl ester, trans-cinnamic acid, and methyl (E)-3′-hydroxyl-4′-methoxycinnamate. El-Sheikh et al. [9] investigated the allelopathic potential, antimicrobial activity, and phytochemical profile of *Artemisia monosperma*, extracts of which impeded the growth of *Chenopodium murale* and *Amaranthus viridis*, while the effects on the crop plants *Solanum lycopersicum* and *Cucumis sativus* were variable. Hofmann et al. [10] reported on the bacterial breakdown products of *Camelina sativa* glucosinolates. These toxic breakdown products can eliminate plant-growth-promoting soil microorganisms and inhibit the pathogen *P. aurantiogriseum*. When combined with glucosinolates, diketopiperazine cyclo(L-Leu-L-Pro), released by the fungus, is fatal to *Camelina* under P-deficiency conditions, as an important *Camelina* root-associated microbial consortium for phosphate solubilization is eliminated in this setting.

Following the path set by the first edition, the second edition of this Special Issue deepens our understanding of allelopathy, thereby helping to foster eco-friendly agroecosystems, protect natural habitats, and preserve biodiversity. To unlock allelopathy’s full potential in regenerative agriculture, we advocate a multidisciplinary approach—spanning chemistry, genetics, metabolism, ecology, and agronomy—that pairs rigorous laboratory screening of novel allelopathic species and their key allelochemicals with robust field trials. These on-farm studies, often conducted using allelopathic cover crops in rotation, should track impacts on soil health, weed control, and subsequent cash-crop performance. By bridging lab assays and real-world systems, this Special Issue aims to translate proofs of concept into sustainable agricultural practices.
